# CT Scan Screening for Lung Cancer: Risk Factors for Nodules and Malignancy in a High-Risk Urban Cohort

**DOI:** 10.1371/journal.pone.0039403

**Published:** 2012-07-02

**Authors:** Alissa K. Greenberg, Feng Lu, Judith D. Goldberg, Ellen Eylers, Jun-Chieh Tsay, Ting-An Yie, David Naidich, Georgeann McGuinness, Harvey Pass, Kam-Meng Tchou-Wong, Doreen Addrizzo-Harris, Abraham Chachoua, Bernard Crawford, William N. Rom

**Affiliations:** 1 Division of Pulmonary, Critical Care, and Sleep Medicine, Department of Medicine, New York University School of Medicine, New York, New York, United States of America; 2 Division of Biostatistics, Department of Environmental Medicine, New York University School of Medicine, New York, New York, United States of America; 3 Division of Thoracic Imagining, Department of Radiology, New York University School of Medicine, New York, New York, United States of America; 4 Division of Thoracic Surgery, Department of Cardiothoracic Surgery, New York University School of Medicine, New York, New York, United States of America; 5 Division of Medical Oncology, Department of Medicine, New York University School of Medicine, New York, New York, United States of America; Clinica Universidad de Navarra, Spain

## Abstract

**Background:**

Low-dose computed tomography (CT) for lung cancer screening can reduce lung cancer mortality. The National Lung Screening Trial reported a 20% reduction in lung cancer mortality in high-risk smokers. However, CT scanning is extremely sensitive and detects non-calcified nodules (NCNs) in 24–50% of subjects, suggesting an unacceptably high false-positive rate. We hypothesized that by reviewing demographic, clinical and nodule characteristics, we could identify risk factors associated with the presence of nodules on screening CT, and with the probability that a NCN was malignant.

**Methods:**

We performed a longitudinal lung cancer biomarker discovery trial (NYU LCBC) that included low-dose CT-screening of high-risk individuals over 50 years of age, with more than 20 pack-year smoking histories, living in an urban setting, and with a potential for asbestos exposure. We used case-control studies to identify risk factors associated with the presence of nodules (n = 625) versus no nodules (n = 557), and lung cancer patients (n = 30) versus benign nodules (n = 128).

**Results:**

The NYU LCBC followed 1182 study subjects prospectively over a 10-year period. We found 52% to have NCNs >4 mm on their baseline screen. Most of the nodules were stable, and 9.7% of solid and 26.2% of sub-solid nodules resolved. We diagnosed 30 lung cancers, 26 stage I. Three patients had synchronous primary lung cancers or multifocal disease. Thus, there were 33 lung cancers: 10 incident, and 23 prevalent. A sub-group of the prevalent group were stable for a prolonged period prior to diagnosis. These were all stage I at diagnosis and 12/13 were adenocarcinomas.

**Conclusions:**

NCNs are common among CT-screened high-risk subjects and can often be managed conservatively. Risk factors for malignancy included increasing age, size and number of nodules, reduced FEV1 and FVC, and increased pack-years smoking. A sub-group of screen-detected cancers are slow-growing and may contribute to over-diagnosis and lead-time biases.

## Introduction

Over 94 million current and former smokers are at increased risk for lung cancer, and might benefit from an effective screening test for early detection. [1], [2] The challenge is to develop a screening strategy that will identify a small lung nodule, and specify whether the nodule is malignant before micro-metastasis occurs [3], [4]. Using data from Surveillance, Epidemiology and End-Results (SEER) Registries, estimates are that 70% of lung cancer deaths could be averted if the cancer was diagnosed at an early stage [5]–[7].

The evidence from chest computed tomography (CT) screening studies conducted since the 1990’s show that the scans can detect early lung cancer, but at the cost of false-positive nodules in more than 25% of those screened [8]–[12]. Most studies have been non-randomized, so demonstrating a mortality benefit of CT screening has been difficult. Most recently, however, the National Lung Screening Trial (NLST) found that screening with low-dose chest CTs can decrease lung cancer mortality by 20% compared to chest radiograph screening [13]. However, over 24% of screens were positive for non-calcified nodules (NCNs); more than 96% of these were found to be false-positive results on follow-up.

If we can develop an improved risk-profile to guide CT screening protocols–both to refine who should be screened and to determine which patients with nodules should be followed most closely–we could reduce the cost and anxiety associated with CT screening, by decreasing the need for follow up CTs and by reassuring patients at decreased risk of malignancy. In 2001 the NYU Lung Cancer Biomarker Center (LCBC) began a lung cancer biomarker discovery and validation project, which included low-dose CT-scans at regular intervals. The project’s goal was to evaluate biomarkers to determine lung cancer risk and to differentiate benign from malignant nodules. The NYU cohort differed from those in previously reported CT screening studies. The population was urban, with a risk of asbestos exposure due to referrals from a local utility workers’ union. Since our study design focused on the collection of material for biomarker discovery, we invited participants to return for screening even if CT-scans identified no suspicious findings or nodules remained stable for more than 3 years. Therefore, we have a prolonged follow-up period, with serial CT-scans for up to 10 years.

In this paper, we review the demographic, clinical, and radiographic characteristics of individuals with nodules on their initial CT compared to individuals with no nodules, and of our patients diagnosed with lung cancer compared to subjects with presumed-benign NCNs. Our hypothesis was that we would identify risk factors for the presence of nodules on screening CT and for the probability that a nodule identified on CT represents an early lung cancer.

## Methods

### Human Subjects and Ethics Statement

The Institutional Review Board (IRB) for NYU Langone Medical Center and the Bellevue Research Committee (BRC) of Bellevue Hospital Center approved the research. All participants signed written consent forms. Between March 2001 and June 2010 the NYU LCBC recruited 1182 study subjects over 50 years of age with more than 20 pack-years of smoking history. Recruitment included mailings to NYU physicians, presentations to the NYU occupational medicine department and the utility workers’ union of Con Edison, advertisements, and distribution of flyers. Individuals with a prior cancer history or history of chemotherapy were excluded, although those with basal or squamous cell carcinoma of the skin were eligible. Clinic visits included a respiratory, medical and exposure questionnaire; collecting peripheral blood samples; forced expiratory spirometry conducted and analyzed according to ATS Standards [14]; induced sputum using aerosolized saline; and low-radiation-dose CT screening. The questionnaire was a validated ATS respiratory questionnaire that included 115 questions on demographic characteristics, tobacco use, occupation and occupational exposures, alcohol use, family history, medical history, and respiratory symptoms.

### Screening CT Imaging and Nodule Follow-up Protocols

During the course of this decade-long study, a number of different Siemens CT scanners were employed. Initially, we used a 4 detector CT-scanner (Siemens Volume-Zoom Multidetector Scanner, Foecheim, Germany). Using low-dose technique (40–80 mAs) 7 mm-thick sections were acquired and then reconstructed every 6 mm, with the additional acquisition of select high resolution 1 mm images through suspected lung nodules. Subsequently, 16 and 64 detector scanners were used, and with the improved scan capability, the format for obtaining scans was changed. In most cases, 5 mm-thick images were acquired and images were reconstructed every 5 mm, using 1 mm collimation. This technique allowed simultaneous prospective reconstruction of contiguous 1 mm images, obviating the need for additional image acquisitions to perform high-resolution detailed analysis of nodule characteristics and calcification. Maximum bidirectional diameters in the axial plane were measured using electronic calipers. Scans were interpreted by thoracic radiologists and, when abnormalities were present, reviewed by a pulmonologist.

Nodule follow-up protocols were modified during the course of the study based on increasing awareness of the need to minimize radiation exposure. If no NCNs were identified, participants were invited to return for repeat screening annually for the first 3 years of the study, and then every 2 years. If new nodules were identified on initial or repeat screening, a standard nodule follow-up protocol was followed. Follow-up was performed initially according to guidelines proposed by the ELCAP study [9], [10]; subsequently, according to the Fleischner Society guidelines [15]. Specifically, non-calcified nodules over 8 mm and suspicious for cancer were referred for immediate clinical evaluation, including possible PET scans, biopsy, resection or further close observation, based on clinical judgment. For nodules 6–8 mm, a follow-up CT scan was performed at 3–6 months, then at 9–12 months, and then at 24 months if no changes were identified. For nodules 4–6 mm, follow-up CT was done at 6–12 months and at 18–24 months if there were no changes. If a solid nodule was stable over time, in size and appearance, for more than 2 years, it was considered benign. Participants were then invited to continue annual or biennial screening. Sub-solid nodules over 6 mm were never classified as benign, and, after 2-year stability, continued CT follow up at 2 year intervals was recommended indefinitely. Since this was a biomarker discovery project, participants were allowed to return to the study at any point, even if they had not followed the recommendations for follow-up.

The radiologists also noted the presence of other radiographic abnormalities. Emphysema was assessed visually as areas of low attenuation contrasting with surrounding parenchyma with normal attenuation. Findings suggestive of asbestos exposure, including pleural thickening or plaques and parenchymal fibrosis consistent with asbestosis (e.g. bilateral, lower lobe irregular opacities), received particular attention.

For each CT scan, the investigators prepared a written report containing detailed radiologic findings and final summary interpretations. CT scan data was entered into the study database by a pulmonologist–listing up to 6 nodules (as per the initial ELCAP definition to define diffuse lung disease), their locations, and for each nodule, solid versus sub-solid opacification. Sub-solid nodules included ground-glass as well as part solid, part ground-glass lesions. The presence or absence of emphysema and other radiographic findings were recorded. The clinical study nurse, the screening coordinator, and the database coordinator kept track of all study participants with non-calcified nodules, and of all lung cancer diagnoses and their follow-ups.

### Statistical Methods

This report is based on two case-control studies. First, to identify risk factors for the presence of nodules on initial screening CT, we compared cases, defined as individuals with NCNs over 4 mm, to controls, defined as high-risk individuals with no nodules on CT-scan. Second, to identify risk factors associated with increased risk of malignancy in patients found to have NCN on screening CT, we compared cases, defined as patients diagnosed with lung cancer, to controls, defined as individuals with presumed-benign NCNs which either resolved or remained stable for more than 3 years. We chose to exclude individuals with sub-solid nodules over 4 mm from the control group, even if they were stable, since we could not be certain that these did not represent malignancy [16], [17]. We did include stable sub-solid nodules less than 5 mm and sub-solid nodules that resolved in the control group.

The factors evaluated included demographic characteristics (age, gender, race, BMI), medical, family and exposure history, respiratory symptoms, CT findings (nodules, emphysema, asbestos-related changes), and PFT results. For each of the variables, we summarized the distributions with descriptive statistics that included mean, median, range and standard deviation for quantitative variables and frequency distributions for qualitative variables. Risk factors associated with nodules versus no nodules and cancers versus benign nodules were identified in separate analyses. Nodules (both solid and sub-solid) were classified based on changes over time (increased, decreased or remained stable). Univariable analyses were conducted to estimate the association of potential risk factors with nodule status and behavior; odds ratios were estimated along with associated 95% confidence intervals and Cochran Mantel Haenszel chi square test statistics were computed. Stepwise polytomous logistic regression methods were used to evaluate the contribution to risk of demographic factors, smoking related factors, and history of other diseases. Stratification was also used to control for possible confounding factors and interaction terms were incorporated. For the lung cancer patients, we used nonparametric comparisons to examine possible differences between cancer patients and individuals with presumed benign nodules.

## Results

### Demographic and Clinical Characteristics at Enrollment

**Figure 1:**
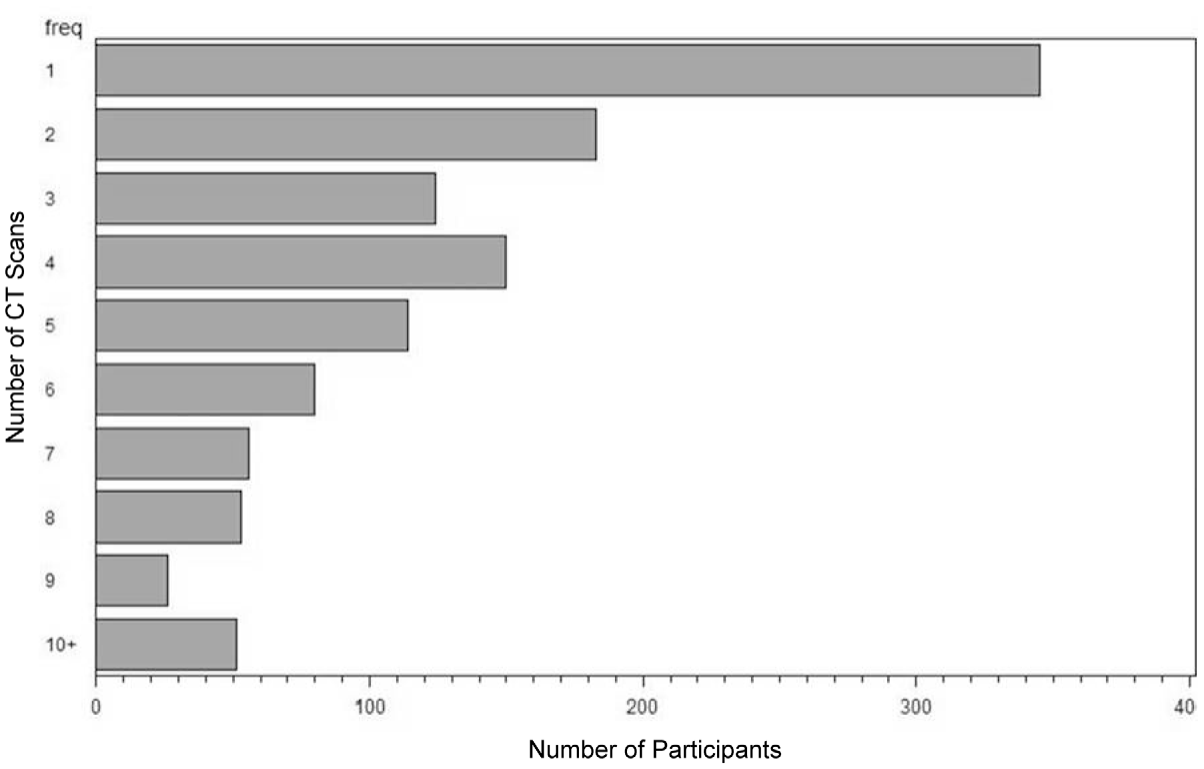
Histogram of the number of CTs performed per individual. Over 70% of participants had at least one follow-up CT. A total of 345 had 1 CT, 183 had 2 CTs and 654 had 3 or more CTs. Those that did not have follow-ups were individuals with no nodules on initial CT who chose not to return or individuals who were not yet due for follow-up by the end of the study period.

All 1182 participants had a baseline CT screen; 819 (70%) had at least one follow-up CT by the end of the study period (Figure 1). Those that did not have follow-ups were individuals with no nodules on initial CT who chose not to return or individuals who were not yet due for follow-up by the end of the study period. The average length of follow up was 43.4 months (range 0–113). The demographics of the screened cohort on enrollment are presented in Table 1. There were approximately equal numbers of men and women, the mean age was 63, and the majority of the participants were white. This finding is partially due to our recruitment from a utility workers’ union, and may also reflect possible barriers to recruitment to cancer-related screening studies among minority populations [18]. Participants were heavy smokers (mean pack-years was 42); more than half of the participants were current smokers at enrollment. Almost 20% of participants had a first-degree relative with a history of lung cancer. Persistent respiratory symptoms were common in this population (Table 1), but pulmonary function was preserved (Table 2). Emphysema was present on the CT-scans of 35.6%.

**Table 1:** Demographic & Clinical Characteristics of the Screened Population at Enrollment.

Characteristic	Number (%)
**Gender**	
Male	616 (52.2)
Female	566 (47.8)
**Age**	
Mean	63±10
>74	158 (13.4)
55–74	825 (69.9)
<55	198 (16.8)
**Race**	
White	1048 (88.7)
Non-white or Hispanic	134 (11.3)
**BMI**	
Mean	27.9±5.4
Underweight (<18.5)	107 (9.1)
Normal (18.5–24.9)	332 (28.1)
Overweight (25–30)	437 (37.0)
Obese I, II, III (>30)	305 (25.8)
**Tobacco Exposure**	
Pack-years	
Mean	42±23
<30	437 (36.9)
30–50	456 (38.5)
>50	291 (24.5)
Current Smoker	651 (55.0)
Second Hand Smoke Exposure	389 (28.9)
**Marijuana Use (ever)**	385 (32.8)
**Occupational Asbestos Exposure**	307 (26.0)
**1st Degree Relative with Lung Cancer**	228 (19.3)
**Persistent Respiratory Symptoms (>3** **mths)**	
Cough	230 (19.5)
Phlegm	220 (18.6)
Dyspnea (*modified MRC scale* *)	
1 (mild)	316 (26.7)
2 (moderate)	99 (8.4)
3 (severe)	37 (3.1)
4 (very severe)	28 (2.4)

* MMRC dyspnea scale: 1 = dyspnea with mild exertion (hurrying on level ground or walking up a slight hill; 2 = dyspnea on level ground walk slower than people of the same age due to dyspnea, or have to stop for breath when walking at usual pace; 3 = dyspnea after walking about 100 yards or after a few minutes on level ground; 4 = too breathless to leave the house or breathless when dressing.

**Table 2:** Pulmonary Function Testing and CT Results of Screened Population.

Pulmonary Function Testing at Enrollment (NHANES)	
FVC	3.72L±1.04
FVC % pred.	93.0%±16.6
FEV1	2.80L±0.83
FEV1% pred.	90.3±18.5
FEV1/FVC	75.0±8.4
**CT Findings**	
Emphysema on initial CT scan	420 (35.5%)
Asbestos-related changes(asbestosis, pleural plaques, and/or pleural thickening)	171 (14.5%)
Nodule findings on first or subsequent CTs	
No nodule	557 (47.1%)
Any nodule >4 mm	625 (52.9%)
Solid nodules only	382 (32.4%)
Sub-solid nodules only	104 (8.7%)
Both SN and sub-solid>4 mm	139 (11.6%)
**Solid Nodules**	
Total individuals with SNs	519 (45% of total)
Solitary	266 (51.3% of SNs)
Multiple	253 (48.7% of SNs)
Total SNs (not individuals) with f/u scans	1084
Resolved on f/u	105 (9.7% of SNs)
Mixed pattern	1 (0.1%)
Decreased on f/u	25 (2.3%)
Increased on f/u	37 (3.4%)
Stable on f/u	916 (84.5%)
**Sub-Solid Nodules**	
Total individuals with sub-solid nodules	239 (20.3% of total)
Solitary	138 (57.9% of SSNs)
Multiple	
Total sub-solid nodules with f/u scans	442
Resolved on f/u	116 (26.2% of SSNs)
Mixed pattern	3 (0.7%)
Decreased on f/u	40 (9.1%)
Increased on f/u	42 (9.5%)
Stable on f/u	241 (54.5%)

Note: many patients had more than one nodule.

Abbreviations: FVC = forced vital capacity, FEV1 = forced expiratory volume in one second, SSN = Sub-solid nodule; SN = solid nodule; f/u =  follow up.

There were 201 individuals referred from Con Edison, most of whom had direct occupational or nearby bystander asbestos exposure based on their job descriptions. Of these, 65 had pleural plaques, pleural thickening, and/or fibrosis consistent with asbestosis (bilateral lower lobe irregular opacities), confirming significant exposure. There were 213 non-Con Edison referrals who also reported significant occupational asbestos exposure; 106 of these had CT-scan changes consistent with exposure. Thus, 171 individuals (14.5% of our total) had confirmed asbestos exposure based on both employment history and CT changes consistent with exposure (Tables 1 and 2). Given the difficulty of assessing actual asbestos exposure, this group with both occupational history and CT changes was the group we considered to have confirmed significant asbestos exposure.

### Nodules

More than half (52.7%) of the participants had NCNs over 4 mm on either their initial or follow-up CT-scans (Table 2). There were 382 individuals (32.4%) with one or more solid nodules; 104 (8.7%) with one or more sub-solid (ground-glass or mixed) nodules; and 139 (11.6%) with both solid and sub-solid nodules. Of the individuals with solid nodules, 51.3% had only 1 nodule. Of participants with sub-solid nodules, 57.9% had only 1 lesion.

We evaluated the solid and sub-solid nodule based on their size and changes over time. We identified 1084 solid nodules in 501 individuals and 442 sub-solid nodules in 213 individuals for whom follow-up CTs were performed by the end of the study period. We classified the nodules as resolved (0 mm for at least 2 consecutive measurements), decreased (decline by at least 3 mm on subsequent CTs), stable (less than 3 mm change on subsequent CTs) or increased (increase by at least 3 mm on follow-up CTs) (Table 2). Sub-solid nodules were more likely than solid nodules to change in size (44.8% versus 15.4%). By the time of the last follow-up included in this study, 9.7% of solid nodules and 26.2% of sub-solid nodules had resolved in 76 and 73 individuals, respectively. A smaller number of nodules had decreased in size, but did not resolve. There were 37 solid nodules in 34 individuals (3.4% of solid nodules) and 42 sub-solid nodules in 27 individuals (9.5% of sub-solid nodules) that increased in size. Some of these were referred for surgery (see below); others are still under close observation due to patient preference for a more conservative approach. The majority of nodules remained stable: 84.5% of solid nodules and 54.5% of sub-solid nodules.

### Lung Cancer Diagnoses

We diagnosed 33 lung cancers in 30 patients. Three patients had more than 1 site of malignancy–considered synchronous primaries or multifocal disease. Twenty-three cases were prevalent at the initial screen and 10 were incident lung cancers identified at the follow up screens. Seven patients underwent surgery for suspicious nodules that were not non-small cell or small-cell lung cancer. The diagnoses included: granuloma, hamartoma, organizing pneumonia, mesothelioma, gastrointestinal tumor metastasis, benign carcinoid and B cell non-Hodgkin’s lymphoma.

The mean age of the lung cancer patients was 69 years, and included 11 men and 19 women (Table 3). They had a mean of 56.5 pack-years of smoking, and 17 were current smokers. Nine patients had a history of significant occupational asbestos exposure (average of 32 years of exposure) and 4 had asbestosis and/or pleural plaques on CT-scan. The majority (65%) of patients had persistent respiratory symptoms; one half had emphysema on their CT-scans. One-fourth had a first-degree relative with a history of lung cancer. Most (28/30) patients had multiple nodules on their CT-scans. Nineteen cancers presented as sub-solid and 14 presented as solid nodules. The average size of the nodules was 10.8 mm on initial CT, and 18.6 mm at diagnosis. The histological types of cancer were 26 adenocarcinomas, 2 squamous-cell carcinomas, 3 small-cell carcinomas, and 2 undifferentiated non-small cell carcinomas. More than 80% of the cancers were diagnosed at stage I. There was no association between the appearance (solid versus sub-solid) of the nodule and the pathologic type, stage at diagnosis or exposure history.

**Table 3:** Lung Cancer Cases Compared with All Screened.

	Lung Cancers	All Screened
	(n = 30 pts, 33 cancers)	(n = 1182)
Mean Age	68.6 (range 53–79)	63
Gender	19 (63%)Female	47.8%F
BMI	27.6 (±6.5)	27.9
Mean Pack Years	56.5 (±20.1)	42 (±23)
Current Smokers	17 (56.7%)	55.0%
Second-hand Smoke	9 (31%)	29.0%
Marijuana Use (ever)	7 (24.1%)	32.8%
Occupational Asbestos Exposure	9 (30%)	26.0%
Asbestosis or Pleural Plaques	4 (13%)	23.5%
1^st^ Degree Relative w/Lung Cancer	7 (24.1%)	20%
Emphysema on CT	15 (51.7%)	35.6%
Respiratory Symptoms >3 months		
Cough	12 (40%)	19.5%
Phlegm	9 (30%)	18.6%
Dyspnea	19 (63.3%)	40.6%
PFT Data		
Mean FVC	3.21±0.8 (89.9% pred.)	93.0% pred.
Mean FEV_1_	2.29L±0.65 (84.3% pred.)	90.3% pred.
Mean FEV_1_/FVC	71.7±9.89	75±8.4
CT appearance of nodules		
Sub-solid	19 (57.6%)	243 pts (20.6%)
Solid	14 (42.4%)	521pts (44.1%)
Pathology		
Adenocarcinoma	26*	
Squamous Cell	2	
Small Cell Lung Cancer	3*	
Non-Small Cell NOS	2	
Stage at Diagnosis		
IA	25	
IB	2	
IIA	1	
IV	4	
Incidence/prevalence	10/23*	
Mean disease-free survival of 30 patients	45.8 months	

* 3 patients diagnosed with 2 primaries. 2 with synchronous adenoca, 1 with prevalence adenocarcinoma and incidence small cell.

**Figure 2:**
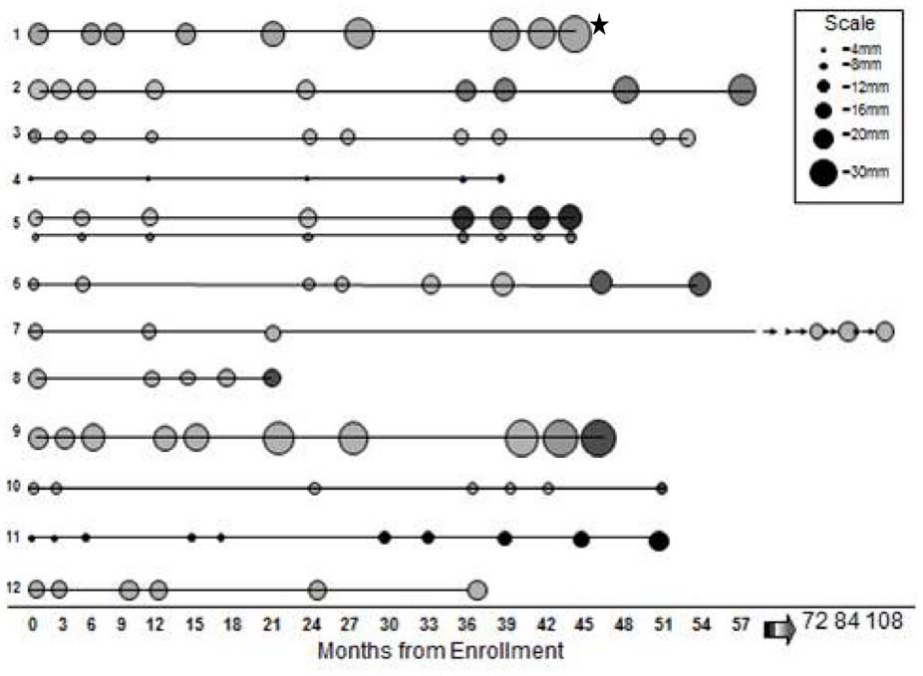
Clinical Course of Prevalent Lung Cancer Cases Diagnosed After More Than 6 Months of Monitoring. Graphic representation of the course of 13 lung cancer cases in 12 patients, plotted by months from enrollment. Each circle represents a point in time relative to enrollment when a CT scan was performed. Surgical resection was performed after the last point in each case. The size of the circle is proportional to the size of the nodule on CT scan. The shading indicates the density of the nodule: grey represents ground-glass density; darker shading represents mixed density and black represents more solid density. 12/13 cases were adenocarcinomas. The asterisk indicates the only patient in this group who has expired due to lung cancer (patient 1).

We categorized cancers as incident if the nodule was not present on the baseline CT-scan, and prevalent if a nodule observed at baseline CT was eventually diagnosed as malignant. Twenty-one patients (23 cancers) had nodules on their initial CTs that were found to be cancer (Table 4). Six of these were diagnosed after identification of a suspicious nodule more than 1 cm in size on the initial CT; 4 were diagnosed after the first follow-up CT for a suspicious nodule seen on the baseline CT demonstrated growth. Twelve patients (13 cancers) had nodules on their initial CT that were followed, remained stable for varying lengths of time, and then were noted to increase in size or density (Table 4 and Figure 2). All but one of these cases were stable for more than 3 years prior to diagnosis. The course of these apparently more indolent cases is shown in Figure 2∶7 nodules increased in size, 1 increased in density, and 5 increased in both size and density. Three of the patients initially refused surgery; 3 had negative PET scans and were followed. The prevalence cases diagnosed after >6 mth follow-up were initially smaller than the prevalent cases diagnosed within 6 months (mean 12.8 mm versus 19.1 mm), and were followed for a mean of 50.3 months and underwent an average of 7.8 CTs. Eleven out of 13 of these cases presented as ground-glass nodules, 12 out of 13 were adenocarcinomas.

**Table 4:** Characteristics of Prevalent versus Incident Lung Cancers.

	Size & CT Densityat Enrollment	Size & CT Densityat Diagnosis	Mean Time Priorto Diagnosis	Histology	Stage at Diagnosis	Median Disease-Free Survival After Diagnosis,& Outcomes
**All cancers (33) (includes** **3 synch. primaries)**	10.8 mm9 SN 14 SSN	18.6 mm19 SN 14 SSN	39.8 mths	26 adeno2 squamous3 small cell2 NSCLC	26 Stage I2 Stage II4 stage IV	42 (range 6–113) mths5 expired (lung ca.)4 recurrences (local)1 lost to f/u
**Prevalence cancers dx** **<6** **mths (10)**	19.1 mm7 SN3 SSN	21.6 mm7 SN3 SSN	2.4 mths	9 adeno1 NSCLC	7 Stage I2 Stage II1 Stage IV	81.5 (11–113) mths2 expired (lung ca.)1 recurrence (local)
**Prevalence cancers dx** **>6** **mths (13)**	12.8 mm2 SN11 SSN	20.1 mm7 SN6 SSN	50.3 mths	12 adeno1 squamous	13 stage I	47 (5–96) mths1 expired (lung ca.)3 recurrences (local)1 expired (cva)
**Incidence cancers (10)**	Not presenton initial CT	13.6 mm5 SN5 SSN	63.5 mths	5 adeno1 squamous3 small cell1 NSCLC	6 stage I3 stage IV	13 (6–33) mths2 expired (lung ca)1 lost to f/u

* Numbers equal 33 cancers in 30 patients, since three patients had more than one diagnosis of lung cancer (synchronous or second primaries).

Abbreviations: adeno = adenocarcinoma, NSCLC = non-small cell lung cancer not otherwise specified, SN = solid nodule, SSN = sub-solid nodule, ca. = cancer, cva = cerebrovascular accident.

Ten patients had incident cancers that developed during the course of the study (Table 4). The average period of observation prior to the appearance of the nodule was 63 months. The average length of time between a prior CT and appearance of the malignant nodule was 13.8 months (range 6–24). Some of these patients were being followed for other nodules; some were undergoing repeat annual or biennial screening CTs. The mean size of the nodule at diagnosis was 13.6 mm. All the cases of small-cell lung cancer were diagnosed as incident cases.

There was no association between the appearance of the nodules (solid versus sub-solid), or the overall outcome and whether the cancer was incident or prevalent or diagnosed before or after 6 months of follow-up (Table 4). Although overall survival was not significantly different, local recurrences and second primaries occurred in 3/12 individuals in the >6 mth prevalence group. There is not as long a follow-up period for many of the incident cases, but the data suggests that these are more aggressive cancers.

### Models for Assessment of Nodules–two Case-control Studies

We compared individuals with nodules on their initial CT with individuals who had no nodules on any of their CTs, and then examined associations of the presence of a solid or sub-solid nodule with patient characteristics. In these polytomous logistic regression analyses, increasing age, male gender, and history of emphysema were the only significant predictors of the presence of a solid or sub-solid nodule (Table 5). When we stratified by gender, in men, older age was a risk factor for the presence of both solid and mixed nodules; the presence of emphysema was a risk factor for solid nodules. In women, the only association found was between older age and the presence of mixed nodules. Of note, smoking history (years of smoking, cigarettes smoked per day, pack-years, second-hand smoke exposure and current smoking status), asbestos exposure and presence of respiratory symptoms were not significantly associated with nodule class in our multivariable analyses.

**Table 5:** Odds Ratios for Risk Factors for the Presence of Nodules on Initial CT Scan*.

	Parameter	Category	OR	95% OR Limit-Lower	95% ORLimit-Upper	p-value
**All participants**	**Gender** **(female vs. male)**	SSN	0.50	0.31	0.80	0.004
	**Age** **(older vs. younger)**	Mixed	1.06	1.04	1.09	<.0001
	**Emphysema** **(present vs. absent)**	SN	1.50	1.10	2.03	0.01
	**Emphysema** **(present vs. absent)**	SSN	1.68	1.05	2.70	0.03
**Men**	**Age (older vs. younger)**	SN	1.03	1.01	1.05	0.011
	**Age (older vs. younger)**	Mixed	1.07	1.03	1.10	<.0001
	**Emphysema (present vs. absent)**	SN	1.97	1.31	2.95	0.001
**Women**	**Age (older vs. younger)**	Mixed	1.06	1.03	1.09	0.0002

* Stepwise logistic regression, with OR calculated by gender, presence of emphysema or current age versus 1 year increase in age. For example: OR shows increased age to be a significant risk factor for nodules versus the reference parameter, younger age. Only significant predictors for each category are included.

Abbreviations: SN = solid nodule, SSN = sub-solid nodule.

We compared the individuals diagnosed with cancer (n = 30) to those with only presumed-benign nodules (n = 128 subjects) to identify risk factors for nodules that may represent lung cancer (Table 6). Presumed-benign nodules were defined as solid or sub-solid nodules that resolved on follow-up and solid nodules that decreased in size or remained stable for more than 2 years. We excluded persistent sub-solid nodules from this group, since we and others [16], [17] have found that a significant percentage of sub-solid or ground-glass nodules represent malignancy. As noted above, 11 of our lung cancer cases presented as ground-glass nodules that remained stable for more than three years prior to diagnosis. Some individuals had multiple nodules–the benign nodule group had only presumed-benign nodules.

**Table 6:** Lung Cancers Cases versus Participants with Benign Nodules: Comparisons at initial Visit.

Variable	Lung cancer cases(n = 30)	Subjects with Benign nodules* (n = 128)	P-value**
Mean FVC% predicted	89.9	96.5	0.0129
Mean FEV1% predicted	84.3	93.9	0.0068
Mean FEV1/FVC	71.7	75.7	0.1498
Emphysema	52%	43%	0.3932
Mean Pack years	56.5	40.0	<0.0001
Current smoker	56%	55%	0.3619
Mean # of solid nodules	2.2	1.5	0.032
Mean # of sub-solid nodules	3.1	0.2	<0.0001
Mean total number of nodules	5.3	1.7	<0.0001
Mean size of nodules	8.1	6.3	P<0.001

* Benign nodules  =  solid nodule stable >2 yrs, or any nodule (solid or sub-solid) that resolved.

** Average scores were used for ties, Kruskal-Wallis test.

The FVC and FEV_1_ percent-predicted were significantly lower and the mean pack-years smoking was significantly higher for cancer subjects than for individuals with benign nodules. The ratio of FEV_1_/FVC was also lower, and there was more emphysema on CT-scan in the lung cancer patients, although these differences were not statistically significant. Compared with individuals with benign nodules, individuals with lung cancer more frequently had multiple nodules (average total number 5.3 versus 1.7), which tended to be sub-solid density (average number of sub-solid nodules 3.1 versus 0.2) and larger in size (average size 8.1 mm versus 6.3 mm). No significant association was found for the other variables analyzed, which included age, gender, BMI, family cancer history, asbestos exposure, and respiratory symptoms.

## Discussion

As part of a lung cancer biomarker epidemiological and validation center, we followed 1182 individuals at high-risk for lung cancer due to smoking and other exposures with serial CT-scans over a period of 10 years. We found that a high percentage (52%) of our participants had NCNs, and we diagnosed 30 cases of lung cancer in subjects enrolled over this 10-year period. We conducted two case-control studies: first, a comparison of individuals with NCNs to those without nodules, and second, a comparison of patients diagnosed with cancer to individuals with benign nodules. Risk factors for the presence of NCNs on initial screening CT were older age, male gender, and emphysema (in men). When we compared lung cancer patients and individuals with presumably benign nodules, we found that lung cancer patients had significantly more smoking history, lower FEV_1_ and FVC, and had more and larger NCNs–especially sub-solid nodules. Our lung cancer cases had more emphysema than the group with no nodules, but not more compared to the benign nodules group. Although asbestos exposure was not associated with an increased risk of nodules, 9 of 30 patients with lung cancer had asbestos exposure for a mean of 32 years and 4 had CT-scans with asbestos-related changes. The great majority of the nodules in our study remained stable or resolved, and only a small percentage (3.4% of solid nodules and 9.6% of sub-solid nodules) grew.

We diagnosed 23 prevalent cancers and 10 incident cancers. Of the prevalent cancers, 12 were present on initial CT, but remained stable for over 3 years prior to the diagnosis of lung cancer. These cases were all stage I, and 11 were adenocarcinomas. The stage at diagnosis and outcome did not differ in this indolent-prevalent group compared with the standard prevalence and incidence groups. This data suggests that some nodules considered benign after several years of stability on CT-scan are malignant, and that delayed diagnosis may not affect outcome. Of the 10 incident lung cancers, 9 were diagnosed after more than 3 years of CT-scans without suspicious findings.

CT screening trials worldwide have encountered the same issues: large numbers of non-specific nodules are discovered, necessitating repeated CTs or invasive procedures [10], [19]; and it has also been difficult to demonstrate a mortality benefit due to the non-randomized nature of most trials, and the possibility of over-diagnosis of indolent cancers [20], [21]. Japanese investigators pioneered low-dose CT population screening. Studies in Japan of CT screening have reported overall cancer detection rates of about 0.4%, with about 90% of the cancers detected at Stage 1 [22], [23]. In these studies, the risk factors for malignant outcome of ground-glass nodules were increased initial size, part-solid appearance, and a family history of lung cancer [16], [17], [24]. The percent malignancy of the ground-glass lesions ranged from 21%–43% for nodules less than 10 mm. Similarly, CT screening trials in Europe [25]–[31] and the United States [9]–[11], [32]–[41] have reported cancer detection rates from 0.9%–4.7%, and NCN rates from 23% to 66%.

The specter of over-diagnosis and lead-time bias has also haunted CT screening trials. When Bach and colleagues evaluated 3 single-arm CT-scan screening studies from the Mayo Clinic, Milan, Italy, and the Moffitt Cancer Center, they found no evidence of lung cancer deaths being averted [41]. They concluded that screening was detecting slow-growing malignancies resulting in lead-time bias, and that CT screening could not detect the aggressive lung cancers that cause higher early mortality. The recent analysis of a randomized Danish lung cancer screening trial also showed a stage shift with CT screening but no improvement in lung cancer mortality [42]. In contrast, as mentioned, the NLST found that screening with low-dose chest CTs can identify early lung cancers and decrease lung cancer mortality, but at the cost of large numbers of non-specific NCN and a 96% false-positive rate [12].

These findings suggest that while lung cancer CT screening can detect early lung cancer and improve lung cancer mortality, it remains an imperfect method of lung cancer screening. A large number of non-specific NCN are detected, which require follow-up, and a disproportionate number of slow-growing tumors are identified. The challenge therefore, is to develop a screening strategy that will identify those at highest risk of lung cancer, detect small lung nodules in these individuals, specify whether the nodule is malignant, and distinguish those malignant nodules that will progress from those that may be more indolent and could be managed more conservatively.

Analyses of the published trials have tried, as we did, to identify risk factors for lung cancer in the screened populations, and factors that may distinguish malignant from benign nodules. Lindell and colleagues emphasized size, solid or semi-solid appearance, and growth rates as important for lung cancer determination [43]. A Spanish multivariate analysis found that emphysema but not airway obstruction was associated with increased risk of lung cancer [44]. In an analysis of the University of Pittsburgh CT-scan screening cohort, increasing GOLD class was associated with lung cancer, as was radiographic emphysema [40]. An analysis the Italian COSMOS screening trial determined that the strongest predictors of lung cancer risk at screening entry were age and smoking duration and intensity [45]. The strongest predictors of lung cancer after first screening were the presence of emphysema and nodules on initial CT–larger nodule size and non-solid nodule type indicated the highest risk. A meta-analysis in 2008 supported the increased risk of lung cancer in individuals with reduced FEV_1_ [46]. Maldonado et al. found no association between emphysema and lung cancer in a nested case-control study using quantitative emphysema analysis [47]. A recent meta-analysis found that emphysema was associated with increased risk of lung cancer only in studies where the assessment of emphysema was made visually, and not by automated densitometric assessment. [48] This finding may explain some of the conflicting results in the literature regarding emphysema and lung cancer risk. The finding that the presence of multiple sub-solid nodules increases the risk of lung cancer may reflect the increased risk associated with lung scarring [49].

Our study confirms that increased age and emphysema are risk factors for the presence of NCNs on CT-scan. We also found that decreased FEV_1_ and FVC, smoking history and the presence of multiple sub-solid nodules were the strongest predictors that an individual with NCNs will be diagnosed with lung cancer. This data can be used to begin to refine the ideal population for screening. Age, smoking history, and presence of emphysema should all be considered when deciding whether to recommend lung cancer screening. After a small non-specific NCN is identified on initial screening CT, reduced lung function, (particularly FEV1 and FVC), increased smoking history, sub-solid or mixed density of the nodule and increased size or number of nodules increase the possibility that the nodule may be malignant, and should mandate closer monitoring.

Because of the design of our study, we followed individuals with no nodules or stable nodules for a period of up to 10 years. This prolonged follow-up led to additional insights into the issues of over-diagnosis and lead-time bias in lung cancer screening. More than 1/3 of the cancers we diagnosed were indolent adenocarcinomas that remained stable on CT scan for more than 3 years, and were still stage I at diagnosis. Early diagnosis of these indolent cancers may not affect mortality–the perceived improved survival may reflect over-diagnosis of indolent non-lethal cancers or lead-time bias due to the earlier diagnosis of cancers that would have been diagnosed at an early stage anyway. The other striking finding of our study was that all but one of the incident cancers were diagnosed after more than 3 years of normal screening CTs. Based on the NLST, the current recommendation is to perform 3 yearly CTs in high-risk individuals. Our findings suggest that this recommendation may be too short.

As CT screening for lung cancer becomes widespread, we still face many uncertainties. The optimal CT screening schedule, both to follow NCNs and to monitor high-risk individuals without suspicious findings, remains to be defined. Will early diagnosis of the indolent cases change the prognosis? Improved risk stratification will address some of these issues. A breath or blood biomarker, such as a panel of protein markers, auto-antibodies, microRNAs or methylation patterns, would also be useful in, defining the nature of indeterminate NCN and distinguishing the more aggressive from the indolent lung cancers [50].
